# A versatile real-time vision-led runway localisation system for enhanced autonomy

**DOI:** 10.3389/frobt.2024.1490812

**Published:** 2024-12-06

**Authors:** Kyriacos Tsapparellas, Nickolay Jelev, Jonathon Waters, Aditya M. Shrikhande, Sabine Brunswicker, Lyudmila S. Mihaylova

**Affiliations:** ^1^ School of Electrical and Electronic Engineering, University of Sheffield, Sheffield, United Kingdom; ^2^ Windracers, University of Southampton Science Park, Southampton, United Kingdom; ^3^ Distributed Avionics, University of Southampton Science Park, Southampton, United Kingdom; ^4^ Research Center AIDA^3^ (AI for Digital, Autonomous and Augmented Aviation), Purdue University, West Lafayette, IN, United States

**Keywords:** aerial systems: perception and autonomy, vision-based navigation, computer vision for automation, autonomous landing, autonomous vehicle navigation

## Abstract

This paper proposes a solution to the challenging task of autonomously landing Unmanned Aerial Vehicles (UAVs). An onboard computer vision module integrates the vision system with the ground control communication and video server connection. The vision platform performs feature extraction using the Speeded Up Robust Features (SURF), followed by fast Structured Forests edge detection and then smoothing with a Kalman filter for accurate runway sidelines prediction. A thorough evaluation is performed over real-world and simulation environments with respect to accuracy and processing time, in comparison with state-of-the-art edge detection approaches. The vision system is validated over videos with clear and difficult weather conditions, including with fog, varying lighting conditions and crosswind landing. The experiments are performed using data from the X-Plane 11 flight simulator and real flight data from the Uncrewed Low-cost TRAnsport (ULTRA) self-flying cargo UAV. The vision-led system can localise the runway sidelines with a Structured Forests approach with an accuracy approximately 84.4%, outperforming the state-of-the-art approaches and delivering real-time performance. The main contribution of this work consists of the developed vision-led system for runway detection to aid autonomous landing of UAVs using electro-optical cameras. Although implemented with the ULTRA UAV, the vision-led system is applicable to any other UAV.

## 1 Motivation

In recent years, UAVs have increasingly been included in the realm of automation due to their ability to mitigate or remove possible human errors when it comes to performing monotonous, perilous and time-consuming tasks. Computer vision and UAVs are unified in a system, to be employed in different applications, including agriculture (Perz and Wronowski, 2018), terrain modelling and map modelling (Zongjian, 2008), cross-regional logistics (Windracers, 2023), and robot-assisted landing (Maier et al., 2016). UAVs are contributing to several different applications by improving safety assurance, increasing operational efficiency and mitigating the effects of human fatigue and error, especially in monotonous and repetitive tasks. Some critical functionalities required of UAV swarms are collision avoidance, risk mitigation in the event of a collision as well as safe landing. The paper proposes a system for detecting the runway sidelines using vision-based methodologies for automating the landing procedure.

Niu et al. (Niu et al., 2022) proposed a landing approach on mobile UGV by using quick response (QR) codes that indicated different altitudes. The approach adopted by the proposed UAV landing system is based on the identification of the runway target to extract a region of interest (ROI) using feature-matching of multiple images and sidelines prediction using probabilistic methods which leads to decision-making for abort or landing.

The main contributions of this paper are the following.

• This paper presents a novel real-time vision-led runway detection framework that adopts feature-matching, specifically the Speeded-Up Robust Features (SURF) (Bay et al., 2006) for runway region extraction. It includes the fast, versatile Structured Forests (Dollár and Zitnick, 2013) for edge detection, combined with Kalman filtering for smooth prediction of the runway sidelines (Borkar et al., 2009; Deng, 2020).

• A comparison of the proposed framework for runway sideline detection with the well-established edge Canny edge detection algorithm is carried out. High accuracy is demonstrated on simulated and real-world videos from the biggest fixed-wing UAV built so far in the UK (Windracers, 2023).

• A comprehensive evaluation of the developed system is performed on varying lighting and weather conditions. These include clear weather, fog, rain, low visibility and crosswind landing. The system performance is validated over videos from the X-Plane 11 simulator (Research, 2017) and real flight video data collected with the ULTRA UAV (Windracers, 2023).

**FIGURE 1:**
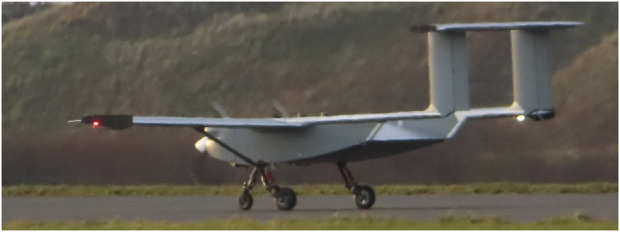
ULTRA self-flying cargo UAV (Windracers, 2023), one of the biggest UAVs in the UK, capable of carrying 100 kg of payload.

The structure of the paper is as follows. The related work summarized in Section 2 presents vision systems that use well-established computer vision approaches as well as deep learning. The related work is focused on previous systems for runway identification and edge extraction systems. Section 3 describes the architecture of the proposed vision-led system for runway detection and sidelines tracking. This section also presents the overall system architecture, and its links with the autopilot and ground control system. Section 4 presents our results and the evaluation of the impact of changing reference image quantities on processing time and accuracy. This section also compares the edge detection algorithms and shows the results of the comparison based on manually generated ground truth data from the ULTRA UAV (Windracers, 2023) shown in Figure 1. The setup used for the experiments is also described in Section 4. Finally, Section 5 summarizes the results.

## 2 Related work

### 2.1 Vision-based automated UAV landing systems

Many approaches to autonomous landing assume that UAVs are equipped with a Global Navigation Satellite System (GNSS) module (Abbott and Powell, 1999), (Patrik et al., 2019). However, UAVs often operate in GNSS denied environments. Additionally, the downside of depending on GNSS is its unreliability for runway alignment during the landing approach since it does not provide heading measurements and the GNSS is vulnerable to local electromagnetic interference. Furthermore, an operator planning the mission far away from the runway will often rely on digital maps which may not always be accurate. Therefore, even with perfect GNSS signal reception, the UAV may attempt a landing offset from the runway.

Runway detection is crucial for such a system. Amit et al. (Amit and Mohan, 2021) proposed a runway identification and tracking model with vision-based methodologies, processing and identification. Akbar et al. (Akbar et al., 2019), assign template matching, Hough Transforms (Cantoni Virginio et al., 2013), active contours and machine learning into two groups, the template matching and feature-based approaches. The author proposes a low-cost solution to detect landing sites by providing rich textual information based on the features. The authors of (Magallán-Ramírez et al., 2022) have utilised a combination of Canny edge detection filters and Hough Transforms for the purposes of mapping for a ground-based robot operating in a maze.

Liu et al. (Liu et al., 2018) proposed a sensor-based, real-time runway detection system. The system uses a search region, and the runway template is generated using topological and sensory data from their proposed “Synthetic Vision System” (SVS) and “Enhanced Vision System” (EVS). The identification of the runway area is based on template matching.

Jbara et al. (Abu-Jbara et al., 2015) proposed a system for runway detection and tracking. The system can be implemented for automatic takeoff and landing for UAVs. Segmentation region completion and reduction of energy function were used for runway edge identification in the video. Cortés-Pérez and Torres-Méndez (Cortés-Pérez and Torres-Méndez, 2021) adopt an approach where they use a Kalman Filter for generating robust measurements of the position of the object of interest even if it leaves the field of view. A more pertinent application of this is illustrated in (Borkar et al., 2009), wherein a Kalman filter is used to update the runway position, which uses sensor data and vehicle attitude. This enables robust tracking and estimation of runway sidelines.

Nazir et al. (Nazir et al., 2018), use images from an airborne camera upon which they employ edge detection algorithms for localising the runway. This paper proposes an evaluation method based on identification and classification and a comparison of the processing times of different runway identification models.

### 2.2 Distinction between traditional computer vision methods and deep learning

Airport detection is a task that was featured in (Chen et al., 2018), using Faster R-CNN (Ren et al., 2015). Numerous deep-learning methodologies have been published, that partially solve the task of runway detection. Such an example is the line-segment detector used as proposed in (Zhang et al., 2017) for classification over the regions.

Akbar et al. (Akbar et al., 2019), proposed a system that has two stages for feature extraction on images and classification. CNN is used for feature extraction and a softmax layer as a classifier for runway identification. Hough transforms (Cantoni Virginio et al., 2013) are used to extract the contours of the lines for runway segmentation.

Traditional computer vision methods rely on hand-crafted features and rule-based systems to analyse images and videos. These methods often require a significant amount of domain knowledge and can be prone to errors in certain scenarios (Mohd Razak and Jafarpour, 2020; Aliyu et al., 2020). For example, traditional computer vision algorithms designed to recognize objects in images can fail when presented with images taken from different angles or under different lighting conditions (Litjens et al., 2017). Additionally, traditional methods may not be able to handle a wide range of images and videos, making them less flexible than deep learning-based approaches (Mohd Razak and Jafarpour, 2020; Aliyu et al., 2020).

Deep learning models promise higher accuracy than traditional approaches if a sufficient amount of training data is available (Sinha et al., 2018; Balduzzi et al., 2021). By providing more data to these models, the performance can be improved and can be adapted for a wide range of scenarios (Yuan et al., 2020). A downside of such models is the big data requirement for training as shown in (Sinha et al., 2018) and the computational resources needed for on-line inference. These may impact the suitability for real-time applications on systems with limited computational power.

The next subsection gives a succinct overview of feature-based approaches for runway detection.

### 2.3 Feature-based approaches

**FIGURE 2:**
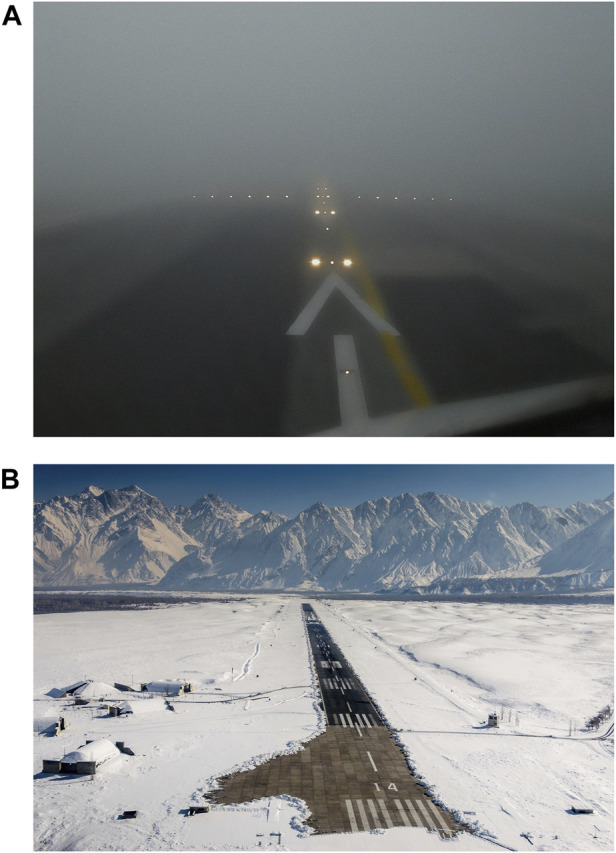
Runways on weather with fog and snow.

Feature-based approaches, as outlined by Lowe (Lowe, 2004), operate without being tied to a specific model for detecting and tracking corners, edges, and other easily localized elements and can instead track custom and complex features. The cost reduction in the model creation is an advantage of this approach in comparison with template matching. It exhibits resilience in adverse weather conditions, such as low visibility due to fog or snow. In such conditions certain runway features may be obscured, potentially causing inaccuracies in detection, as illustrated in Figures 2A, B.

Even in challenging weather conditions, these feature-based techniques, including well-established ones like SIFT (Lowe, 2004), SURF (Bay et al., 2008), and edge detection (Sharifi et al., 2002), remain widely applied in autonomous systems.

The SIFT algorithm (Lowe, 2004; Zhang, 2022) is invariant to rotation, distortion, translation, noise and change in illumination and is chosen for features extraction from images. The invariance properties of the SIFT algorithm make it a viable candidate for intelligent flight tasks.

In the context of runway landing approaches, Miller et al. (Miller et al., 2008) leverage SIFT to detect terrain as visual information. This strategic use allows the UAV to navigate toward the runway even before it becomes visible by registering images against prior images in which the location and orientation are known.

However, the computational time and accuracy of the original SIFT algorithm as evaluated by Daixian (Daixian, 2010), makes it unsuitable for real-time applications.

Consequently, to address these concerns an enhanced version of SIFT has been put forth, demonstrating improved real-time performance, algorithmic stability, and matching accuracy.

Bay et al. introduced Speeded-Up Robust Features (SURF) (Bay et al., 2006), an efficient image descriptor that outperforms state-of-the-art methodologies. The SURF descriptor is based on sums of Haar wavelet components and is asserted by the author to be an efficient and effective scale and rotation-invariant interest point detector and descriptor. The SURF algorithm outperforms the histogram-based techniques utilized in the SIFT algorithm.

The basic procedures of SURF resemble those of SIFT (Lowe, 2004). In the publication of Bay et al. (Bay et al., 2008), three primary processes are used for identifying discrete image point correspondences, namely,: the selection, feature vector computation and the matching. During the selection process, the interest points are selected such as corners, and T-junctions. The feature vector that is calculated represents the neighbourhood of each interest point. The final stage is the comparison and matching of images based on the computed feature vectors.

SURF, as described in the article of Bay et al. (Bay et al., 2008), is based on the Hessian filter rather than relying on a histogram of locally oriented gradients near the key point like SIFT algorithm. The detector being employed here is based on the SURF algorithm. Box filters and integral image results in the filter size replace the SIFT technique of down-sampling by scaling up rather than scaling down when transitioning between different scale spaces. According to Bay et al. (Bay et al., 2008), this adjustment is anticipated to potentially lead to an increase in computational performance.

SURF has been proven to be more efficient than SIFT with respect to the matching speed (Bay et al., 2008). A variety of research was conducted on SURF, by different researchers on different applications, to verify high performance and robustness, Liu and Wang (Liu and Wang, 2009) and Vardhan et al. (Vardhan et al., 2015). Applications in which the SURF algorithm can be used to utilise the increased computational performance are synthetic aperture radar (SAR) image matching (Liu and Wang, 2009), visual tracking (Li et al., 2012), and face recognition (Gupta et al., 2021).

## 3 A Synergistic approach for runway detection

### 3.1 Region of interest extraction

The speed advantage of SURF can be attributed to many factors in its feature extraction process including its use of a pre-computed integral image. An integral image represents a type of data-structure called summed-area table, which allows for fast evaluation of the sum of values in a rectangular subset of a grid (Viola and Jones, 2001). It significantly speeds up calculations. The integral image II is computed by:

$$II\left(x,y\right)=\underset{x\leq x^{\prime },y\leq y^{\prime }}{\sum }i\left(x^{\prime },y^{\prime }\right)$$ (1)

where, $\left(x^{\prime },y^{\prime }\right)$ denotes a pixel in the original image, (x,y) denotes a pixel in the integral image, $i\left(x^{\prime },y^{\prime }\right)$ is the intensity value of the pixel $\left(x^{\prime },y^{\prime }\right)$ and II(x,y) is the intensity of the integral image at pixel (x,y) .

The integral image (Equation 1) (Viola and Jones, 2001) can be computed efficiently in a single pass using the equation:

$$II\left(x,y\right)=i\left(x,y\right)+II\left(x-1,y\right)+II\left(x,y-1\right)+II\left(x-1,y-1\right)$$ (2)

Once the integral image (Equation 2) has been computed, the evaluation of the sum of values within a rectangular sub-region in the image requires only four array references to the integral image, regardless of the size of the sub-region. For a rectangular sub-region denoted as ABCD where $A\left(x_{0},y_{0}\right)$ , $B\left(x_{1},y_{0}\right)$ , $C\left(x_{0},y_{1}\right)$ and $D\left(x_{1},y_{1}\right)$ are the vertices of the rectangular sub-region, the sum of values within this sub-region can be calculated by:

$$\underset{x_{0}\leq x\leq x_{1},y_{0}\leq y\leq y_{1}}{\sum }i\left(x,y\right)=II\left(D\right)+II\left(A\right)-II\left(B\right)-II\left(C\right),$$ (3)

where $x_{0},y_{0}$ and $x_{1},y_{1}$ are the respective coordinates of the considered vertices in the rectangular sub-region.

The use of integral images guarantees that the summation of pixel values in Equation 3 is done in constant time, thus delivering speedy results regardless of the size of the input image.

The SURF algorithm (Bay et al., 2006) involves several key equations, including the creation of a Hessian matrix (Equation 4), descriptor computation (given below with Equation 6), and orientation assignment (Equation 5).

The Hessian matrix is computed using box filters:

$$H\left(x,y,\sigma \right)=\left[\begin{array}{cc} L_{xx}\left(x,y,k\sigma \right) & L_{xy}\left(x,y,k\sigma \right)\\ L_{xy}\left(x,y,k\sigma \right) & L_{yy}\left(x,y,k\sigma \right) \end{array}\right],$$ (4)

where $L_{xx}$ , $L_{xy}$ , and $L_{yy}$ are second-order partial derivatives, k is a constant determining the scale and σ is the standard deviation of the Gaussian kernel. Orientation $\theta \left(x,y\right)$ is assigned based on the dominant direction of the local image gradient:

$$\theta \left(x,y\right)=\arctan\left(\frac{L_{xy}\left(x,y,\sigma \right)}{L_{xx}\left(x,y,\sigma \right)}\right).$$ (5)

The SURF descriptor is computed using Haar wavelets and the following equation:

$$D_{i}=\underset{x,y}{\sum }\omega \left(x,y\right)\cdot h_{i}\left(x,y\right),$$ (6)

where $h_{i}\left(x,y\right)$ represents Haar wavelet responses, and ω(x,y) is a Gaussian weighting function.

The SURF algorithm is faster at feature extraction than SIFT due to its use of integral images, box filters, Hessian matrices, Haar wavelets and a lower number of scales in its scale-space representation. Given the computational speed benefits of SURF over SIFT as demonstrated in (Bay et al., 2008; Liu and Wang, 2009), SURF is chosen as the feature extraction algorithm in the proposed method. The output of the SURF algorithm is a set of keypoints and an N x128 array of keypoint descriptors, where N is the number of keypoints found in the image, and 128 is the number of descriptors for each keypoint.

In the proposed implementation, SURF is used to extract features and keypoints from the current video frame as well as from the reference images. The algorithm iterates within the dataset of reference images and finds the best matching image, depending on the number of keypoints successfully matched between the current video frame and the reference image. This feature matching is performed using OpenCV (Bradski, 2000) functions.

Using built-in OpenCV functions, the homography matrix between the best matched reference image and the current video frame is calculated. The in-image locations of all the matched keypoints from both the images are used to calculate the homography matrix between the two images. This homography matrix is then used to transform the bounding box of the runway Region Of Interest (RoI) from the reference image to the current frame. The runway RoI thus derived for the current frame is then fed to the edge detection block of the vision system.

### 3.2 Object tracking

Object tracking algorithms can be used as a tool for minimising the computation time in cases where a system sequentially performs feature matching on the frames with a feature matching algorithm. This can exponentially increase the processing time in exchange for negligible increases in accuracy. A relevant example of combining feature matching and object tracking is the derivation of the region of interest using SURF and tracking the region of interest using object tracking in consecutive frames. Object tracking can be applicable in various autonomous systems, where the target object is identified, and a region of interest is outlined through a bounding box and needs to be tracked for a number of frames.

The tracker is defined as the motion model that tracks the speed and direction of the object’s movement, and appearance in the frame (Sarkar et al., 2022). The Channel Spatial Reliability Tracker (CSRT), as noted in the comparative study (Sarkar et al., 2022) demonstrates high efficiency, accuracy and versatility and is adopted within the proposed approach for tracking the runway RoI.

CSRT as implemented in OpenCV is based on the paper by Lukezic et al. (Lukežič et al., 2018) that utilises Discriminative Correlation Filters (DCFs) (Hester and Casasent, 1980) enhanced with spatial and channel reliability for performing robust real-time object tracking. The advantage with this approach is that it does not require the tracked object to be of rectangular shape unlike other trackers (Lukežič et al., 2018) and can drive the object search towards areas in the image that have a higher probability of containing that object. It also delivers robust tracking performance even in the event of occlusion, background clutter and non-rigid transformations of the object.

DCFs work by using a set of filters to correlate a template of the target object with a search window in subsequent frames to find the location of the target object. The CSRT (Lukežič et al., 2018) takes this a step further by implementing spatial and channel reliability in the filtering step to allow for robust tracking even in case of occlusions. It uses different feature channels like Histogram of Oriented Gradients (HOG) and color histograms and assigns weights to each channel to denote their reliability in effectively defining the object. It also implements spatial reliability which allows the tracker to focus on parts of the object that are more reliable for tracking. The spatial reliability map is constructed from the output of a graph labelling problem wherein pixels or patches of the image are treated as nodes on a graph and relationships between the nodes are represented as edges. These learned relationships allow the preservation of spatial information and allow the tracker to learn local as well as global information about the target. This helps in tracking even in the event of occlusion.

With DCFs, the goal is to learn a filter w which produces strong responses at the in-image target location when correlated with input sources, and produces weak responses everywhere else. Given a set of training examples { $x_{i}$ } and corresponding labels { $y_{i}$ }, the filter w is learned by minimising the following objective function:

$$L\left(w\right)=\underset{i}{\sum }\|w*x_{i}-y_{i}{\|}^{2}+\lambda \|w{\|}^{2},$$ (7)

where * denotes a convolution operation, $y_{i}$ is the Gaussian-shaped label centered at the target and λ is a regularisation parameter. The ‖.‖ denotes the Euclidean norm operation.

The channel reliability feature is achieved by optimising the objective function

$$L\left(w^{c},\alpha_{c}\right)=\underset{i}{\sum }\|\alpha_{c}\left(w^{c}*x_{i}^{c}\right)-y_{i}{\|}^{2}+\lambda \|w^{c}{\|}^{2}.$$ (8)

A filter is also implemented with a matrix of coefficients $w^{c}$ for each feature channel c , with $\alpha_{c}$ denoting the channel reliability weight. Here, $x_{i}^{c}$ denotes the feature map of channel c for the $i^{th}$ sample. The spatial reliability map M is learned adaptively, modifying the objective function to:

$$L\left(w^{c},\alpha_{c},M\right)=\underset{i}{\sum }\|\alpha_{c}\left(w^{c}*\left(M⊙x_{i}^{c}\right)\right)-y_{i}{\|}^{2}+\lambda \|w^{c}{\|}^{2},$$ (9)

where ⊙ denotes element-wise multiplication. The filter and weights $W^{c}$ are updated iteratively

$$W^{c}=\frac{\underset{i}{\sum }X_{i}^{c}⊙\bar{Y_{i}}}{\underset{i}{\sum }X_{i}^{c}⊙\bar{X_{i}^{c}}+\lambda }.$$ (10)

where $X_{i}^{c}$ denotes the Fourier transform of $x_{i}^{c}$ , $\bar{Y_{i}}$ denotes the Fourier transform of $y_{i}$ and $\bar{X_{i}^{c}}$ denotes the complex conjugate of $X_{i}^{c}$ . The filter weights update (Equation 10) typically involves solving a ridge regression problem in the Fourier domain due to the convolution operation (Liu and Dobriban, 2019). Thus, in each frame, the response map is computed by applying the learned filter, with main Equations 7–10 to the current frame’s features and the position with the new response is considered as the target’s new position. The filter and reliability maps are updated periodically to adapt to changes in the target’s appearance. Since the CSRT is much faster, it allows the vision system to keep track of the runway RoI without having to run the SURF feature extraction on every frame.

### 3.3 Structured forests for fast edge detection

The structured forests dedicated to fast edge detection can be considered as a computer vision methodology that was proposed by P. Dollár and C. L. Zitnick, (Dollár and Zitnick, 2013). The structured forests algorithm includes learning by generating decision trees for the local structures in the ground truth annotated images. Critical attributes of the training are the orientation, color, texture and gradient magnitude. The resultant structured forests are used on new images to predict edges during the testing process rapidly. A key advantage of the structured forest algorithm (Dollár and Zitnick, 2013), is its computational efficiency, which is important for real-time applications.

The algorithm aims to learn a map denoting the feature space of the input image. The individual nodes of the decision trees that are constructed during the training process predict a local structure. Significant attributes used for training include gradient magnitude, orientation, color, and texture features (Dollár and Zitnick, 2013). The edge prediction efficiency is achieved thanks to the aggregation of the outcomes from all decision trees.

Since the edge detection block detects edges in all orientations in the image, a Hough transform (Cantoni Virginio et al., 2013) is used to truncate horizontal edges and only retain edges with orientations between [π/4,3π/4] and [−π/4,−3π/4] . The rationale behind this is that when the aircraft is on approach for landing, the runway edges will not exceed a 45deg inclination with respect to the x -axis.

### 3.4 Runway sidelines prediction and tracking

The output of the Hough transform consists of a set of lines represented by polar coordinates in the form ( r , θ ), where r is the length of the perpendicular line connecting the coordinate origin and the Hough transform line and θ is the orientation of that perpendicular line with respect to the x -axis. Next, a Kalman filter (Borkar et al., 2009) is designed to predict the location of the two sidelines of the airport runway.

The Kalman filter gives the predicted distance r and the predicted orientation angle θ and their predicted derivatives $\dot{r}$ and $\dot{\theta }$ of the left (L) and right (R) runway sidelines. The state vector is represented as: $x={\left[r_{L},\dot{r_{L}},\theta_{L},\dot{\theta_{L}},r_{R},\dot{r_{R}},\theta_{R},\dot{\theta_{R}}\right]}^{T}$ . Here ^T^ denotes a transpose operation. The Kalman filter uses as measurements the information from the edge detection block which consists of the Hough transform results (Cantoni Virginio et al., 2013). The Kalman filter state update is based on a constant velocity model, with a unit sampling time. The measurement update equation is also a linear model, with a unit measurement matrix.The system state and measurement noises are considered to be mutually uncorrelated, white noises. The state covariance matrix Q is in the form: $Q=diag\left(Q_{L},Q_{R}\right)$ , with $Q_{L}=Q_{R}=diag\left(0.25,0.5,0.5,1\right)$ and the measurement matrix is: R=I∗0.9 and I the identity matrix. The values of Q and R are chosen based on physical considerations linked with the prediction error and inaccuracies in the video data, respectively. Accurate prediction of sidelines is achieved in real-time using the frames based on variance and noise.

### 3.5 System architecture

The proposed system is a combination of the approaches so far illustrated. The main assumption here is that the camera is centrally mounted on the UAV such that a central line drawn in the video frame accurately denotes the centre line of the UAV. The core approach adopted for deriving the region of interest that describes the runway is the SURF algorithm, used for performing feature matching (Bay et al., 2008). The second process being performed is lane detection and tracking in the predicted runway region. The lane prediction is performed using a combination of Structured Forests edge detection, Hough transforms and Kalman filtering (Borkar et al., 2009).

Whilst in simulation mode, live feed video that simulates a flight scenario is transmitted from the main computer. The main computer acts as the camera of the system that runs the X-Plane 11 flight simulator alongside the autopilot controlling the mission and the video server that is responsible for transmitting the video throughout the network. The onboard software is hosted on the development board which includes the software that detects the runway, predicts the lanes and makes a decision of landing or abort.

**FIGURE 3:**
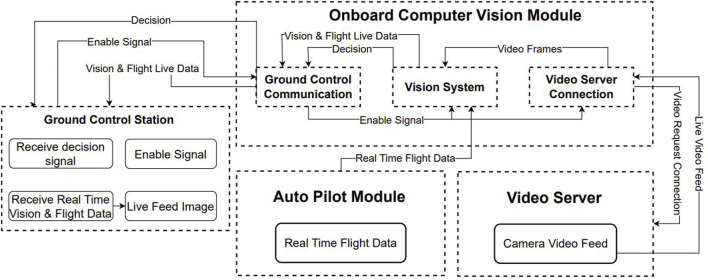
Overall system block diagram.

Upon activation, the onboard system is connected to the autopilot, receiving the mission information such as waypoints, and landing waypoints. A connection to the video server is established during system activation and after mission derivation. The overall approach can be observed in Figure 3. The system then uses the prior knowledge from airport runways at different locations, from previous flights, to extract features for the process of feature matching. The vision-based auto-landing system is armed during the whole flight and it is part of the decision-making process during the UAV landing approach phase.

**FIGURE 4:**
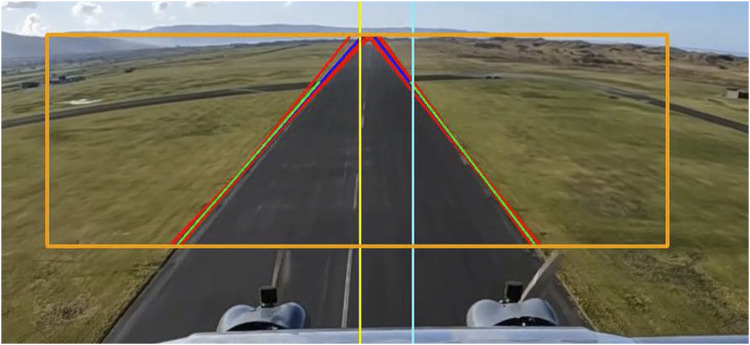
Complete system on real-world scenario.

Once the autopilot conveys that the UAV is on approach for landing, the system enables the automated landing vision software where the live feed images captured from the UAV are processed. Feature matching is applied between the prior knowledge features and the live feed to extract an RoI describing the runway. The RoI is then used to extract the runway’s sidelines. The sidelines are predicted using the Kalman filter at every time step. Figure 4 shows the complete system with the bounding box of the region of interest, the resultant lines from edge detection and Hough transform in red and the merged lines in blue. The green lines represent the predicted lines from the Kalman filter. The runway edges outside the bounding box are not detected since the algorithm performs the search only inside the area of the image enclosed by the bounding box as the bounding box demarcates the runway RoI. This is done to reduce processing times as the size of the image to be analysed becomes smaller. The altitude at which the vision system is triggered is decided based on operational requirements and constraints.

**FIGURE 5:**
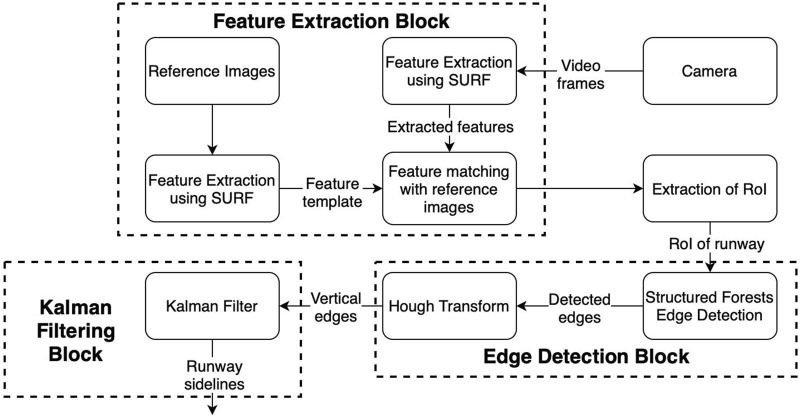
Vision system information flow.

The functioning of the vision system can be observed in Figure 5. The main components of the vision system are: 1) the feature extraction block, 2) the edge detection block and the Kalman filter block (as described in Section 3.5. The raw video frames are fed to the feature extraction block in a sequential manner, where the SURF algorithm is applied to extract features from each frame. These extracted features are then compared to the features extracted from the reference images and template matching is performed. Once the runway location is verified using the template matching, an RoI is then adopted for the video stream. The desired RoI from the input image is then selected and fed to the edge detection block, where the RoI is processed by applying structured forests edge detection. A Hough transform is then applied to the sequential images in order to discard horizontal lines. The output of the Hough transform is then supplied as input to a Kalman filter which provides an efficient prediction of the left and right runway sidelines. The RoI used for the runway is tracked using the CSRT algorithm for a number of consecutive frames, and then an update step of feature matching is executed to update the RoI describing the runway and continue the process of runway line prediction.

### 3.6 Software-in-the-loop simulation

Software-In-The-Loop (SITL) establishes communication between the autopilot module and the X-Plane 11 simulator (Research, 2017), to guide, and extract real-time flight data from the aircraft. The autopilot needs to be configured with the mission that the UAV will follow for the desired flight. Upon configuration and activation, the flight controller will take control of the aircraft and execute the mission from take-off to landing. This methodology allows image extraction and processing during a test flight to be executed in real-time with all the components acting as a closed-loop network.

The closed-loop network setup involves a data link that transmits data from the autopilot module to the computer vision module and the vision module broadcasts that data to the ground station for user observation. The SITL methodology is a crucial part of testing the vision-based system in both simulation and real environments as the system is inactive, deriving images and processing them for runway detection and lane tracking, until the autopilot issues the signal that the UAV initiates the landing approach. The landing or abort decision of the vision-based system will be made at a certain altitude which will be derived from the autopilot.

The SITL is utilized for testing and deployment as it offers ease of deployment with a host machine used as the UAV’s camera, with the processing unit connected directly to that source. This methodology eliminates the necessity of using any hardware dedicated to flight controllers or actual drones, which can be an expensive procedure, especially when running multiple tests over time. The next Section 4 describes the experimental set-up and the evaluation of the results.

## 4 Performance evaluation

### 4.1 Feature matching results

The established system for runway identification and RoI selection relies on feature matching and the SURF algorithm. The selection of SURF was made based on its robustness to distortion, illumination and viewpoint changes. The system activates using the information transmitted by the autopilot module on the landing approach. The system searches for matching features between the reference images and the current live images transmitted by the video server (fixed camera).

The reference images are collected from previous flights of the ULTRA UAV (Windracers, 2023) and datasets were generated for different runway locations. This process was performed manually by an operator using a custom-made user interface which analyses and shows the video to the operator. The operator can stop the video and select the runway. The selected region is then extracted and saved with the airport credentials, for future use in real-time flights.

**FIGURE 6:**
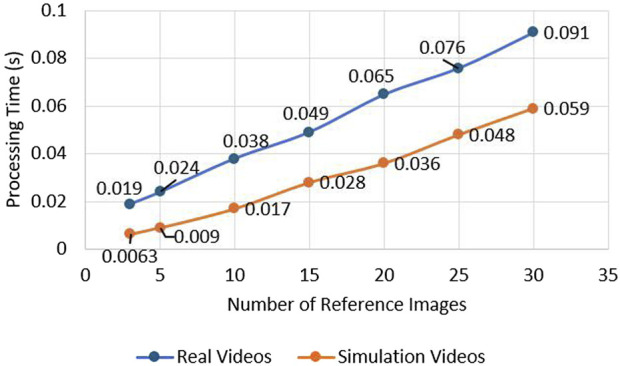
Effect of reference image quantity on navigation system processing time.

**FIGURE 7:**
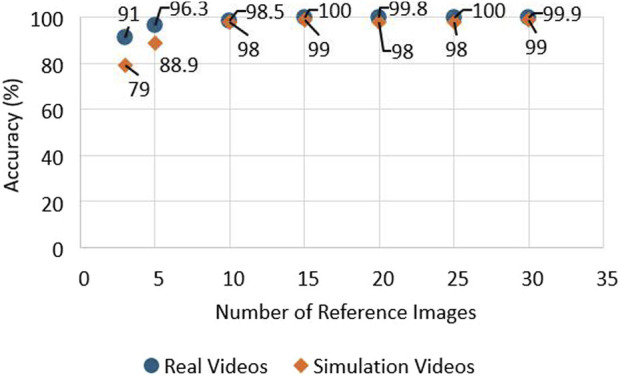
Accuracy impact from image quantity.

The generation of reference image based on prior knowledge is essential for the accurate performance of the system. The operator manually selects images containing the runway RoI which will act as a template for the runway detection system. Reference image datasets were created for several airports. When the vision system is activated, image descriptors and key points are extracted from the reference images. The set of key points is compared with the real-time image. This can be a computationally expensive process when using multiple reference images. An experiment was performed to find the optimal number of reference images so that the computational time is manageable for the system. The results from the testing show that the processing time increases with a larger amount of images as references during the comparison of key points. The second test that was performed is the investigation of how the accuracy of the system on runway identification is affected by the number of images selected as reference (Tsapparellas et al., 2023). The results of the test can be observed in Figure 7. The results show that the accuracy is affected when the reference images are limited from one to nine images. The optimal number of reference images to meet the criteria of high accuracy and fast processing times was decided to be a range from 10 to 15 reference images. These results can be observed in Figures 6, 7.

Additionally, during testing, the approach angle of the aircraft towards landing was seen to have an effect on the efficacy of the runway detection. The vision system was activated halfway down the final approach to the landing in the pre-flare stage (before the aircraft starts pitching the nose up to make contact with the ground). This means that the aircraft was flying parallel to the runway and descending at a constant rate when the vision system was activated. The final approach angle was calculated using the airfield weather conditions to provide a known pitch of the aircraft relative to ground level. The system was tested with different aircraft pitch angles on approach ranging from 4deg to 10deg . At a pitch angle of <6deg , the runway detection and sidelines prediction were unreliable. At pitch angles of >6deg , the runway was consistently detected along with the sidelines. Pitch angles of >10deg were not tested due to the risk of unreasonable downward velocities on landing. This is because a shallow approach angle provides a narrow viewing angle between the camera and the runway and due to the camera placement, the small part of the runway that is visible gets occluded by the aircraft. This could result in insufficient feature extraction by SURF, which in turn could worsen the performance of all downstream image processing blocks. Steep approach angles give a wide viewing angle between the camera and the runway which provides a large view of the runway in spite of the occlusion by the aircraft.

A large view of the runway provides SURF with a good input and results in rich feature extraction and hence good performance by all the downstream components. This problem can be easily remedied by placing the camera on the underside of the aircraft where it can have unhindered views of the runway below.

The one-step ahead predicted sideline parameters are used to calculate the middle line of the runway. Then this information is part of the on-board computer vision module as shown in Figure 4.

### 4.2 UAV flights data repository

In the pursuit of crafting diverse flight scenarios, the X-Plane 11 simulator (Research, 2017), emerges as an advanced flight simulator that offers precise control over aircraft dynamics from take-off to landing. Varied meteorological conditions are used to generate a rich dataset to develop a system with a global solution. X-Plane 11 (Research, 2017) can authentically replicate challenges that a pilot may face during a flight, such as adverse weather. Examples of adverse weather conditions that were simulated are strong winds, fog, and rain.

**FIGURE 8:**
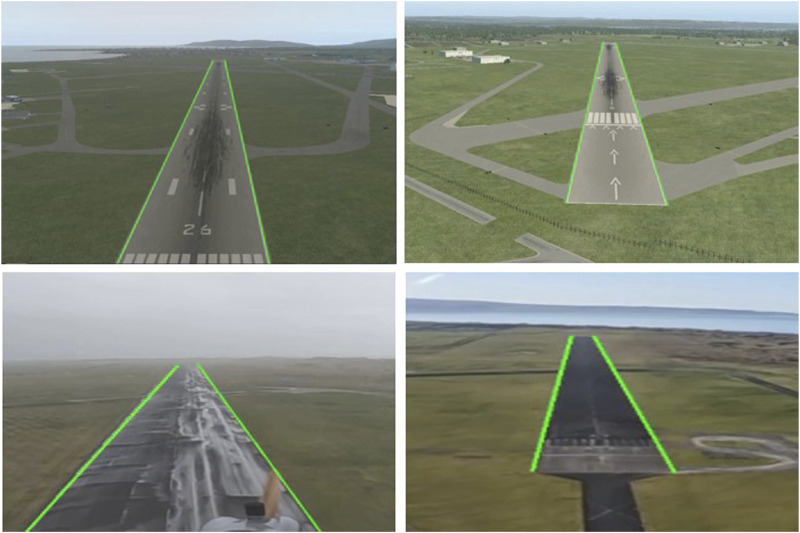
Ground truth example images from the dataset (Tsapparellas et al., 2024).

**FIGURE 9:**
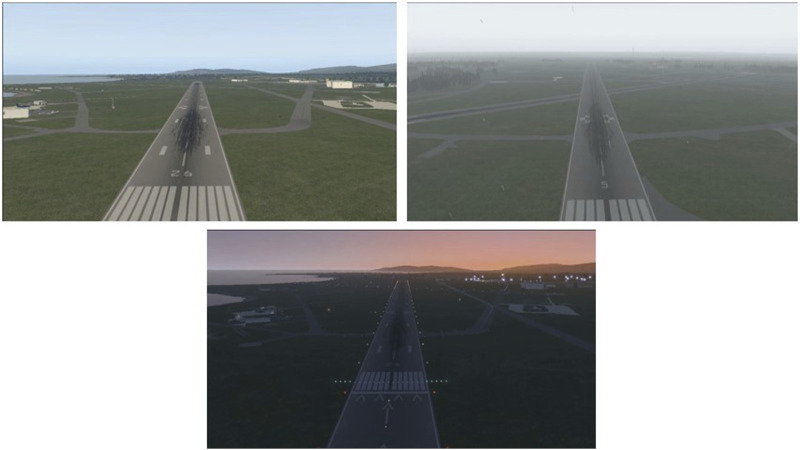
Images from X-Plane 11 simulator from dataset (Tsapparellas et al., 2024).

**FIGURE 10:**
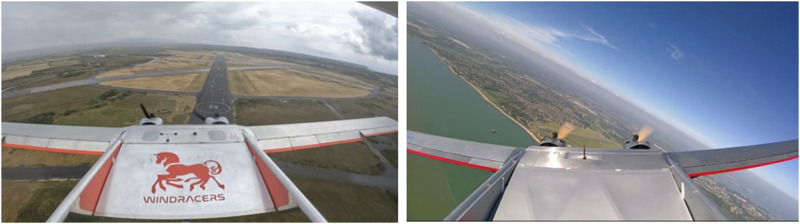
Real images from ULTRA flights from dataset (Tsapparellas et al., 2024).

The ground truth data generated from the simulation environment as well as real flight data is manually annotated. Each frame was manually annotated to describe the sidelines of the presented runway. This experiment will enhance the knowledge of which algorithm performs efficiently and accurately on aerial imaging for the task of automated UAV landing. Examples of the ground truth images can be observed in Figure 8, with the ground truth lines being represented by the light green color. The real-world videos collected with the ULTRA UAV and simulated data, together with the created ground-truth used for validation can be accessed from the University of Sheffield’s ORDA data repository (Tsapparellas et al., 2024). The data includes videos from Solent, Llanbedr airport, Isle of Man and Seattle. The dataset also includes videos generated from the X-Plane 11 Simulator in varying weather conditions such as clear weather, snow and fog, as well as crosswind landing approaches. The repository is a valuable asset for stimulating reproducible research. Figure 9 showcases some example images from the X-Plane 11 simulator and Figure 10 illustrates real images from the flights of the ULTRA UAV. These figures along with Figure 2 showcase the difficulty of the task of runway detection and localisation given the similarity of the runway surface with the surroundings.

### 4.3 Experimental setup

The experimental setup includes the X-Plane 11 simulator and autopilot developed by Distributed Avionics (Avionics, 2023). X-Plane 11 provides an excellent environment for studying different UAV flight phases, including taking off from an airport, circling, cruise, to the approach and touchdown phases - all of which can be compiled into videos for a representation of these diverse scenarios. This approach, inspired by Bittar et al. (Bittar et al., 2014), involves employing a software-in-the-loop system with X-Plane 11 (Research, 2017) and Simulink. Their paper sought to design an algorithm steering a fixed-wing aircraft within the simulation environment. The data collected from this procedure, which is in the form of videos, will be used to test the system in different conditions. The tests were performed on an NVIDIA Jetson Xavier NX development board, which has a 6-core NVIDIA Carmel ARMv8.2 CPU, 8 GB of RAM and a 384-core NVIDIA Volta GPU. The board can draw a maximum power of 20W.

When evaluating the proposed approach against the Canny edge detector, the user provides the region of interest for each step to ensure accurate results of the region of interest extraction, as automating this process is not the experimental objective. The evaluation system then uses the original image as input for both the structured forests model and the Canny edge detector to perform edge detection. The computational time for both methods is measured for all processed frames and it is averaged for each algorithm. The computational time of the proposed intelligent landing approach compared with the state-of-the-art approach is similar, of the order of 0.007 s per frame and a difference of 0.000057 s between the two. This provides real-time performance, with the Canny edge detector being quicker than the proposed approach.

Next, the image Cartesian (x,y) coordinates of the runway sidelines are derived from the predicted Kalman filter states. Since the task is to compare the efficacy of the runway sidelines detection, the predicted lines are compared with ground truth lines generated by the authors. The ground truth lines accurately denote the edges of the runway as seen in Figure 8. Since we are detecting runway sidelines, we end up with two predicted lines, one for each sideline of the runway. We thus have two corresponding ground truth lines and when comparing we must compare each predicted edge to its corresponding ground truth edge, i.e., the left predicted edge must be compared with the left ground truth edge and similarly for the right predicted edge. For this, we must compute the cross product (Equation 11) between a predicted edge and a ground truth edge to see if they correspond to each other in terms of which side of the runway they represent. This cross product is computed as follows:

$$Cross\;product=\left(y_{1}-y_{0}\right)*\left(x_{gt}-x_{1}\right)-\left(x_{1}-x_{0}\right)*\left(y_{gt}-y_{1}\right),$$ (11)

where $\left(x_{0},y_{0}\right)$ and $\left(x_{1},y_{1}\right)$ are points on the predicted line and $\left(x_{gt},y_{gt}\right)$ is a point on the ground truth line. If the value of the cross product is 0, this means that all three points are co-linear and the two lines can be compared. Once it is determined that the ground truth point is co-linear with the predicted line, it is then verified whether the ground truth point $\left(x_{gt},y_{gt}\right)$ lies on the line segment connecting $\left(x_{0},y_{0}\right)$ and $\left(x_{1},y_{1}\right)$ . If both these conditions are satisfied, then the prediction is considered to be a successful one. These condition checks are performed for every predicted line/ground truth line pair and the Accuracy metric (Equation 12) is calculated as follows:

$$Accuracy=\frac{Number\;of\;successful\;predictions}{Number\;of\;total\;predictions}*100$$ (12)

The accuracy metrics can be observed in Table 1. As can be observed from the table, the proposed approach delivers better results in the scenarios with Fog and Crosswind, in Adverse Lighting and delivers better performance on real-world videos. A detailed comparison of edge detection approaches, including recently developed deep learning methods, is presented in the surveys (Zhou et al., 2024; Sun et al., 2022). Deep learning edge detection methods have a potential but they need a significant amount of data and these methods are a scope of a future work.

**TABLE 1:** Accuracy metrics for comparison between the proposed approach using Structured Forests (SF) and an approach using the Canny edge detector. The average results presented in the table are calculated over 3,000 frames.

Runway sidelines detectors	Canny	Proposed SF
Scenario	Accuracy	
Clear Weather Simulation Videos	100%	100%
Fog and Crosswind Simulation Videos	52.25%	56.25%
Adverse Lighting Simulation Videos	87.3%	99.2%
Clear Weather Real Videos	55.3%	82.4%
Overall Information		
Average Accuracy	73.7%	84.4%
Average Processing Time	0.007895	0.007952
Total Frames for Evaluation	3,000	3,000

In addition to the accuracy metric, we have evaluated the F1 score for evaluating the detected lines, following the definition from (Zhou et al., 2024). The F1 score, can be defined as $F1=2N_{c}/\left(N_{g}+N_{t}\right)$ , in which $N_{c}$ , $N_{g}$ , and $N_{t}$ are the number of correct line segment pairs, line segment ground truth, and detected line segments, respectively.

As can be observed in Table 2, the proposed approach delivers a higher ratio of true positives than the Canny edge detector across Fog and Crosswind, Adverse Lighting and on real-world videos, with the highest difference being observed in Adverse Lighting (Simulation Videos) and in Clear Weather conditions (Real Videos).

**TABLE 2:** F1 score metrics for comparison between the proposed approach SF and an approach using the Canny edge detector. The average results presented in this table are calculated over 3,000 frames.

Runway sidelines detectors	Canny	Proposed SF
Scenario	F1-score	
Clear Weather Simulation Videos	0.7898	0.7898
Fog and Crosswind Simulation Videos	0.5218	0.5617
Adverse Lighting Simulation Videos	0.8450	0.9624
Clear Weather Real Videos	0.5044	0.7516
Overall Information		
Average F1 Score	0.6652	0.7663
Total Frames for Evaluation	3,000	3,000

## 5 Conclusion

This paper presents an efficient real-time system for vision-led runway localisation for aiding autonomous landing for fixed-wing UAVs. It can recognise and extract the region of the runway during the UAV landing phase and predicts the side-lines of the runway. A multi-image matching algorithm is used during the runway region extraction and edge detection, which acts as an information bank for the prediction of sidelines using a Kalman filter and smooths the results. Based on the performed experiments, the optimal number of reference images needed to keep high accuracy and low computation time is in the range of 10–15 images. The results from the comparison of edge detectors for accuracy and performance indicate that Structured Forests for edge detection outperform the Canny edge detector on accuracy, with a detection rate of 84.4 % and 73.7 % , respectively. Unlike approaches rooted in deep learning, the proposed approach does not require large amounts of training data and can deliver real-time performance even on low power systems. There is a trade-off in accuracy, speed and robustness when choosing between deep-learning based methods and traditional computer vision methods as seen in the proposed system.

Although the tests were performed on a fixed-wing UAV, the proposed system is generic and can be applied to another type of aircraft. This work paves the route to automating the landing of a single UAV and a swarm of UAVs which is a significant step forward towards improving UAVs autonomy.

## Data Availability

The datasets presented in this study can be found in online repositories. The names of the repository/repositories and accession number(s) can be found below: https://orda.shef.ac.uk/articles/dataset/Data_Repository_from_the_Swarm_of_UAVs_Innovate_UK_Project_Future_Flights_Strand_3_UAV_Flights_Dataset/25712577/1.
